# Back to the future: Using herbarium specimens to isolate nodule‐associated bacteria

**DOI:** 10.1002/ece3.11719

**Published:** 2024-07-14

**Authors:** Renee H. Petipas, Amanda A. Antoch, Ashton A. Eaker, Hanna Kehlet‐Delgado, Maren L. Friesen

**Affiliations:** ^1^ Department of Plant Pathology Washington State University Pullman Washington USA; ^2^ Department of Microbiology University of Washington Seattle Washington USA

**Keywords:** coevolution, herbarium collections, *Medicago lupulina*, nodule microbiome, rhizobia

## Abstract

Herbarium specimens are increasingly being used as sources of information to understand the ecology and evolution of plants and their associated microbes. Most studies have used specimens as a source of genetic material using culture‐independent approaches. We demonstrate that herbarium specimens can also be used to culture nodule‐associated bacteria, opening the possibility of using specimens to understand plant–microbe interactions at new spatiotemporal scales. We used historic and contemporary nodules of a common legume, *Medicago lupulina,* to create a culture collection. We were able to recover historic bacteria in 15 genera from three specimens (collected in 1950, 2004, and 2015). This work is the first of its kind to isolate historic bacteria from herbarium specimens. Future work should include inoculating plants with historic strains to see if they produce nodules and if they affect plant phenotype and fitness. Although we were unable to recover any *Ensifer*, the main symbiont of *Medicago lupulina*, we recovered some other potential nodulating species, as well as many putative growth‐promoting bacteria.

## Graphical abstract

Biological collections have been used to study the genetics and genomics of numerous organisms, including microorganisms. Additionally, herbaria have been used to resurrect extinct and extant plant species. Here we describe methods to resurrect historic microbes from herbarium specimens, allowing for many novel questions to be asked across temporal and spatial scales.

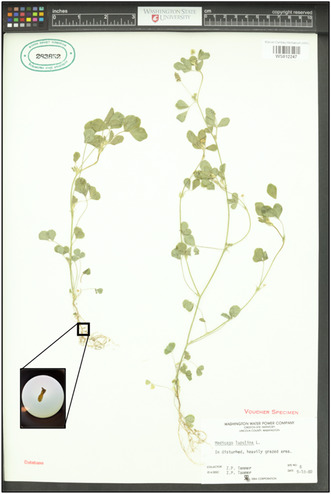

## 1 INTRODUCTION

Herbaria, historically used to answer questions in taxonomy and systematics, are increasingly being used as a resource to investigate questions in genetics/genomics, ecology, biogeography, and evolution. Herbaria have been touted as a potential source of extinct plant germplasm, (Albani Rocchetti et al., 2021, 2022; Wolkis et al., 2022), even in one case allowing researchers to resurrect an extinct species of *Astragulus* (Molnár et al., 2015). With the advent of more sophisticated and increasingly inexpensive molecular methods and a burgeoning interest in microbiomes and plant–microbe interactions researchers are increasing taking culture‐independent approaches to study plant‐associated microbes in botanical collections (Bieker et al., 2020; Campos et al., 2021, 2023; Daru et al., 2018; Heberling & Burke, 2019; Li et al., 2007).

The power of herbarium samples for discovery in plant adjacent fields was evident in a 2001 paper that overturned preexisting beliefs about the origin of the blight that led to the Irish potato famine (Ristaino et al., 2001). Follow‐up work established that historic strains of *Phytophthora infestans* found in herbarium specimens were from an extinct lineage (Yoshida et al., 2013), highlighting that herbaria are a source to discover unknown microbes. Most recent work explored in depth the genomics that lead to the total displacement of historic strains of *Phytophthora* (Martin et al., 2013). Work in wild yam demonstrated, through the generation of 17 historical genomes, a much earlier origin of the obligate symbiont than previously assumed. It also produced evidence of horizontal transmission and gene transfer, which overthrew pre‐existing beliefs in exclusive mode of vertical transmission (Danneels et al., 2021). Even more recently, authors took a metagenomic approach to describe temporal shifts in a common plant species, *Ambrosia artemisiifolia*; this type of work could be critical in expanding our temporal window to understand microbiome shifts in response to anthropogenic activities including climate change (Burbano & Gutaker, 2023).

Less commonly, researchers have taken culture‐dependent approaches. In a recent proof‐of‐concept paper, authors demonstrated that leaf fungal endophytes could be successfully cultured from herbarium specimens (Daru et al., 2018). Similarly, historic tobamoviruses, a stable virion that remains viable for long periods, were used to infect contemporary tobacco plants. Astonishingly, authors were able to infect plants with virus from specimens collected in 1899 and they found no correlation between the age of the specimen and the ability to infect contemporary specimens (Fraile et al., 1997).

**FIGURE 1:**
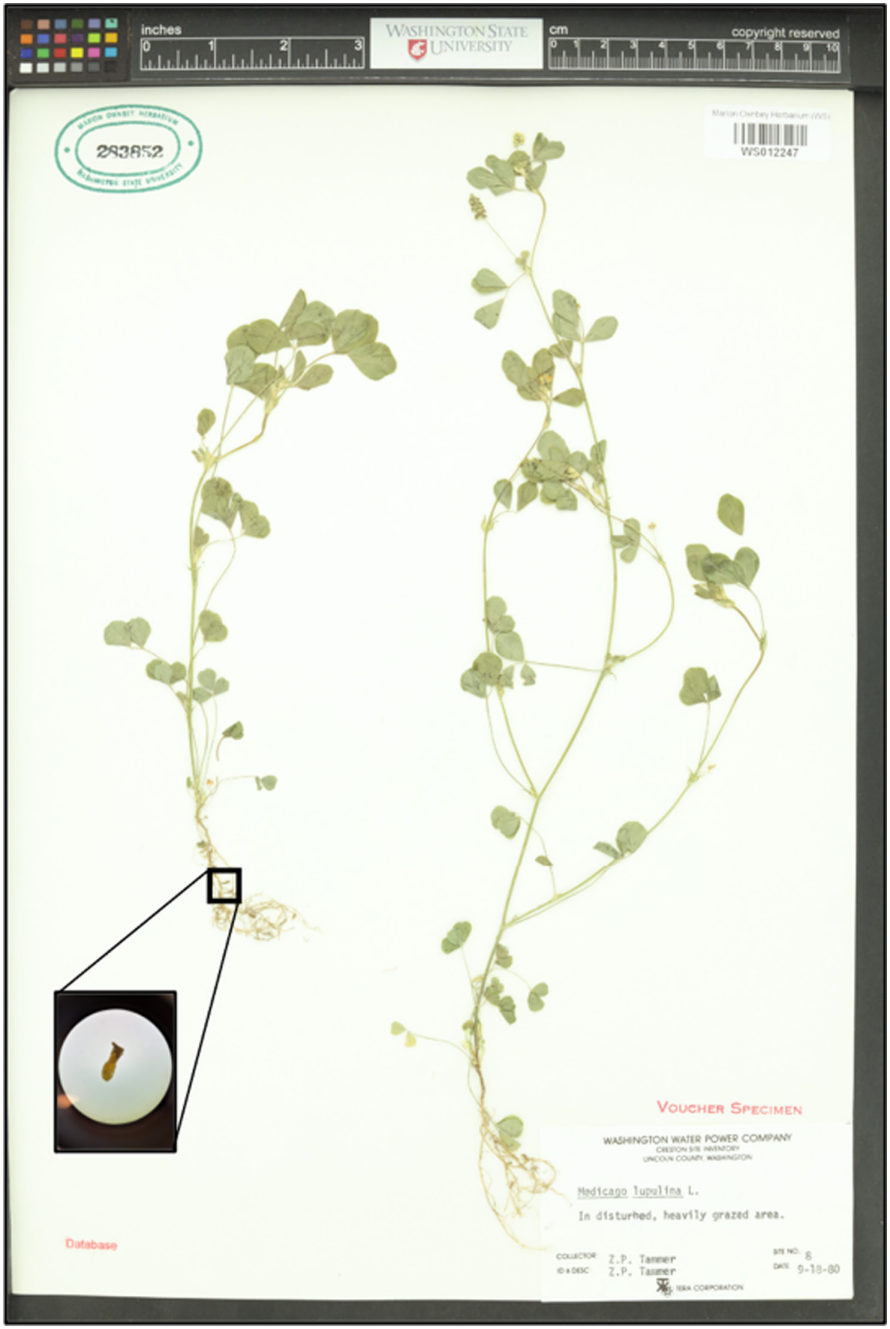
Herbarium specimens are often mounted with roots intact and nodules (inset) can be found on even the oldest specimens. The nodule pictured here was taken from specimen 324,040 collected in 1962 from Walla Walla, Washington. Photo credit: Water Fertig (plant specimen) and Layla Taylor‐Spriggs (inset).

To our knowledge, no one has cultured root‐associated bacteria from herbarium specimens, despite their importance to plant phenotype and fitness (Friesen et al., 2011; Petipas et al., 2020, 2021). Here we work with a common legume species to isolate bacteria associated with contemporary and historic nodules. *Medicago lupulina* is a largely selfing, annual, biennial, or occasionally perennial plant species (Yan et al., 2009) with a wide distribution throughout North America. It was introduced to the continent in the late 1700s (Turkington & Cavers, 1979), and by the early 1800s copious specimens could be found in biological collections throughout North America. Specimens are often mounted with roots intact and nodules can still be found on the roots of even the oldest specimens (Figure 1). *Medicago lupulina* primarily affiliates with two species of rhizobia, *Ensifer meliloti* and *Ensifer medicae* (Harrison et al., 2017).

Besides the main nodule‐forming rhizobia species, nodules are often colonized by a diversity of other bacteria. Work in related *Medicago* species revealed taxa from at least 10 bacterial genera colonizing nodules (Martínez‐Hidalgo et al., 2022). Understanding nodule‐associated bacteria is important because they can perform important functions in the rhizosphere critical to plant development, growth, and the functioning of the legume rhizobia symbiosis. For example, nodule‐associated bacteria can produce siderophores, solubilize phosphate, change root morphology, and enhance nodulation, all of which can lead to enhanced growth and survival in legumes (Martínez‐Hidalgo & Hirsch, 2017). In addition, rhizobia are a phylogenetically diverse group of bacteria, including taxa from *Ochrobactrum* (Trujillo et al., 2005), *Devosia* (Rivas et al., 2002), *Microvirga* (Msaddak et al., 2017), and *Paraburkholderia* (Elliott et al., 2007) are all capable of nodulating plants and fixing nitrogen. This opens up the possibility of finding nodulating rhizobia besides *Ensifer* that nodulate *Medicago*.

Here we used seven media types to isolate bacteria from historic nodules taken from herbarium specimens around Washington State. We were able to culture isolates from three historical specimens, including 13 isolates from a specimen from 1950, 62 isolates from 2004, and 26 isolates from 2015. We sent a subset of sequences for Sanger sequencing and found that we were able to recover a diversity of bacteria including representation from 15 genera. While we were unable to capture any *Ensifer* in our collection we did recover a number of putative plant‐growth promoting bacteria, including rhizobia in the genus *Microvirga*.

## 2 MATERIALS AND METHODS

### 2.1 Herbarium sampling

We used nodules collected from herbarium specimens of *Medicago lupulina* housed at the Marion Ownbey Herbarium at Washington State University. Marion Ownbey houses 400,000 vascular and non‐vascular plant specimens, including 122 specimens of *Medicago lupulina* collected primarily from North America (109 specimens). For our experiments, we used nodules from historic and contemporary specimens collected around Washington State from Skagit County (1 nodule), Klickitat County (2 nodules), Grant County (1 nodule), Yakima County (1 nodule), Ferry County (1 nodule) and Whitman County (2 nodules) from 1925 to 2019 (Table 1). We chose every sample, collected in Washington State, that had roots with nodules. We selected two nodules from the Klickitat County specimen because it had multiple plants on one sheet, and each had nodules. For each specimen, we use a clean, autoclaved razor blade to excise nodules and a similarly sized piece of leaf material to be used as a control for environmental contamination (Heberling & Burke, 2019). We used gloves and a clean surface (ethanol sterilized, autoclaved trays) to collect material. Nodules and leaf material were stored in sterile Eppendorf tubes at room temperature until use in the culturing experiment. We used contemporary nodules collected from the Washington State University campus in Pullman, Washington. Contemporary nodules included one air‐dried nodule, stored at room temp in an Eppendorf tube for 1 year (collected 11/14/2018), a fresh collected nodule (06/06/2019), and a lab strain of *Ensifer medicae* (WSM‐419). Contemporary nodules were collected outside Johnson Hall at Washington State University (46.73138° N, 117.15657° W).

**TABLE 1:** Metadata for the herbarium specimens used in this work, we used nodules from historic *Medicago lupulina* collected from Skagit County (1 nodule), Klickitat County (2 nodules), Grant County (1 nodule), Ferry County (1 nodule), and Yakima County (1 nodule) in Washington State from 1925 to 2015.

Specimen ID	Date collected	Location	Description	Exact date	Notes
338,247	1974	Skagit Co, WA	Burpee Hill above concrete. T35 R8E NW1/4 NW 1/4. 500 ft disturbed clearing at roadside, S facing	3‐Jul‐74	3 specimens on one sheet
390,849	2004	Kickitat Co, WA	Columbia Hills State Park‐Horsethief Lake. Found in area of park closed to public due to cultural resources (Petrogylphs). Growing with *Bromus* and *Boisduvalia*. Petals Yellow. Township 2 North, Range 14 East, Section 19 SE 1/4 of SW 1/4	11‐Jun‐04	3 specimens on one sheet
390,614	2015	Grant Co., WA	Growing in gravel in park in Mattawa. Park is at corner of Portage Avenue and Rd 24 SW. Growing with Trifolium repens and Plantago major. Flowers yellow. Fruits unarmed, Coord: 46.73846, 119.90712	19‐Jun‐15	
305,246	1986	Ferry Co, WA	Kettle Range. Colville National Forest. Thirteen Mile Area. Near end of USFS Road 2055. T35N R34E SW1/4 Sec 19. Elev. 4150 ft. Roadside with Pseudotsuga menziesii, and Larix occidentalis	8‐Jun‐86	2 specimens on one sheet
291,521	1950	Yakima Co, WA	Common on very moist river bottom land, mainly in fence row, approximately 125 yards northwest of the iron bridge, Yakima river, west of Granger. Elevation about 750 feet. Creeping	8‐Jun‐50	2 specimens on one sheet

### 2.2 Culture collection

Seven different media types were prepared: Luria‐Bertani (LB), Tryptone‐Yeast (TY), Yeast Mannitol (YM), YM plus calcium, diluted YM, YM plus sodium chloride (NaCl), and YM plus pyruvate. The YM plus calcium was prepared according to the YM recipe with the addition of 2 grams of calcium carbonate (CaCO_3_) per liter, instead of 1 gram per liter. The diluted YM was prepared as a ½X dilution of YM media for all ingredients except agar. Similarly, the YM plus NaCl was prepared with 1 gram of NaCl per liter, which is 10 times the amount in the protocol. The YM plus pyruvate was prepared as indicated in the protocol, with the addition of 600ul of filter‐sterilized (Acrodisc syringe filter, 0.2 μM, VWR, Atlanta, GA, USA) 2 M sodium pyruvate to the media post‐autoclaving to produce a final concentration of 2 mM sodium pyruvate. Media recipes can be found in Table S1. Luria‐Bertani (LB), Tryptone‐Yeast (TY), and Yeast Mannitol (YM) were all chosen for this study because they are commonly used to isolate rhizobia and non‐rhizobia species in plants. Calcium was added because it has been demonstrated to increase the viability of rhizobia, by strengthening the cell wall (Vincent, 1962). We used a dilute YM in the hopes that by diluting nutrient‐rich media, we might discourage the growth of fast‐growing species such as *Bacillus*, allowing slower‐growing species to grow, as well as lower the presence of free radicals that can inhibit cell growth (Oliver, 2005). Salt was added to retain osmotic balance and regulate membrane potential (Cinátl, 1969). Finally, pyruvate was added to prevent oxidative damage (Giandomenico et al., 1997) by eliminating metabolic by‐products and free radicals (Stevenson et al., 2004).

Nodules and leaves were rehydrated in filter‐sterilized (Acrodisc syringe filter, 0.2 μM, VWR, Atlanta, GA, USA) 10% sucrose solution for 48 h. After rehydration, nodules were rinsed in autoclaved, filtered water Milli‐Q® seven times before transfer to 200ul ½X phosphate‐buffered saline (PBS) for an additional 24 hours. Nodules (and leaves) were crushed and then streaked onto 10 plates of each media type for the 1950 specimen, but three plates of each media type for years 1974–2019. After completing the nodule and leaf control for the 1950 sample it seemed the amount of liquid was not enough to adequately streak 70 plates. An autoclaved, filtered Milli‐Q® water blank and an autoclaved PBS blank were used as negative controls and were streaked onto five plates of each media type. Plates were sealed with parafilm and then incubated at 30°C for 12 h before being observed for growth.

Plates were observed for growth every 24 h. Plates with growth after 5 days were re‐streaked for isolation onto the same media type using the quadrant streak method. Each sample underwent at least two isolation re‐streaks onto the same media type. If a plate had a single isolated strain after two sets of re‐streaks, cells were harvested for glycerol stocks and pellets for DNA extractions. If the plate was not a single isolated strain (>1 colony morphology on the plate), isolation restreaks continued for each morphologically unique colony on the plate until they were isolated. This took up to four sets of re‐streaks for some isolates. We described the isolates using criteria from the American Society for Microbiology (Breakwell et al., 2007), describing the pigmentation, form, margin, elevation, and texture (Table S2). Glycerol stocks were made by flooding the plates with 3 mL of sterile ½X PBS. Cells were lifted from the surface of media and mixed into the buffer using sterile plastic pestles. We added 750 μL of cell/buffer slurry to each 2 mL stock tube each with 750 μL of 80% glycerol. Stock tubes were vortexed and then stored in the −80°C freezer. The remaining cell/buffer slurry was added to a 2 mL Eppendorf tube and centrifuged at 10,000 rpm for 3 min or until a pellet formed. Once pelleted, all supernatant was removed. Pellets were stored at −80°C prior to use for DNA extractions.

### 2.3 Sanger sequencing

We used isolate descriptions to select morphologically diverse isolates to send for Sanger sequencing because we wanted to describe the diversity within our collection. We identified 52 isolates for Sanger sequencing (~48% of our collection) and we performed DNA extractions using the Qiagen PowerLyzer Microbial DNA Kit (Germantown, MD, USA). We then amplified the 16S rRNA region with primers 27F and 1492R, which span nearly the entire length of the gene (Weisburg et al., 1991). Amplicons were prepared with 2 μL of template, 39.75 μL of molecular grade water, 5 μL of DreamTaq® buffer, 1 μL of 10xDNTP mix, 1 μL forward primer (27F), 1 μL reverse primer (1492R), and 0.25 μL DreamTaq®. Thermocycler conditions for bacterial PCR amplification were 95°C for 5 min, followed by 33 cycles of 95°C for 30 s, 53°C for 30 s, 72°C for 2 min, with a final extension of 72°C for 3 min. PCR products were purified using Sera‐Mag Speedbeads carboxylated magnetic beads (Thermo Fisher Scientific, Waltham, MA, USA) and eluted in molecular grade water. Samples were submitted to the Washington State University Laboratory for Biotechnology and Bioanalysis (LBB) for Sanger sequencing using the ABI 3730 DNA Analyzer (ThermoFisher Scientific). Isolates that sequenced poorly were run twice. Forward and reverse sequences from three isolates did not pass quality thresholds and were not included in subsequent analyses.

Sanger sequences were trimmed to “max ambiguities” = 2 and consensus sequences were made by combining forward and reverse reads with Geneious Primer (v. 2023.2.1, Biomatters, Inc., CA, USA) with the “high sensitivity/slow” method. For taxonomic identification, sequences were used as BLAST queries to the NCBI 16S rRNA database with the following settings: “low complexity filter, max e‐value = 0.05, word size = 28, gap cost = linear, program= megaBLAST.” The species with the lowest E value and highest percentage identity were selected as the identity of the isolate. Here we present taxa to the genus level, but putative species IDs can be found in Table S3.

### 2.4 Data analysis

To analyze the probability of growth we used binomial generalized linear models (lme4 package in R, Bates et al., 2015). We used backward model selection to find the minimal model that best explained the data. We began with the full model and sequentially dropped terms until we arrived at a minimal model (Crawley, 2015). Models were compared with likelihood ratio tests and a critical value of 0.05. To estimate the probabilities associated with growth and better understand treatment effects we used the emmeans (Lenth, 2022). Our data analysis sought to answer three questions: (1) Were our negative controls significantly different from plates streaked with historic/fresh nodules? (2) Was the probability of growth from fresh versus historic nodules dependent on media type, and (3) Does media type affect the growth of historic isolates from different years? To analyze question 1, we specified if a sample was a microbiology control, environmental contamination control (leaf), or historic/fresh nodules. The full model included an interaction between treatment (controls vs nodules) and media type. To analyze question 2, we removed negative controls and specified if a nodule was historic or fresh. The full model included an interaction between treatment (historic vs. fresh) and media type. To analyze question 3, we removed fresh nodules. The full model included an interaction between nodule year and media type.

## 3 RESULTS

### 3.1 Culture collection

**FIGURE 2:**
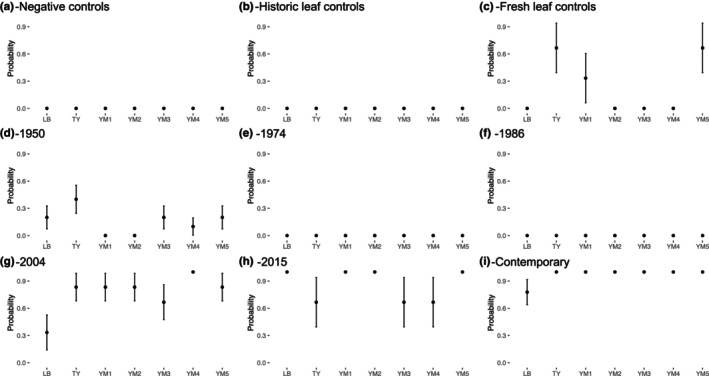
We had no growth on any of our negative controls (a) and historic leaf controls (b). We had leaf endophytes grow from our fresh controls, however only for a subset of the media types (c). We had a low probability of growth for plates streaked with crushed nodules from 1950 (d) and no probability of growth on plates streaked with nodules from 1974 (e) and 1986 (f). The probability of growth was high for more recent specimen nodules including 2004 (g) and 2015 (h). The probability of growth was nearly 100% with contemporary nodules (i). LB, Luria‐Bertani; TY, tryptone‐yeast; YM1, yeast mannitol; YM2, YM + calcium; YM3, Dilute YM; YM4, YM + sodium chloride; YM5, YM+ pyruvate.

To confirm that our isolates cultured from fresh and historic nodules were not the result of contamination from the herbarium or the laboratory, we compared our media blanks and herbarium leaf controls to growth on plates that were streaked with either fresh or historic crushed nodules. We did not find any growth on our negative controls (Figure 2a) or historic leaf samples (Figure 2b), compared to ~50% probability of growth on plates streaked with nodules from historic and contemporary sources (χ^2^ = 177.6, df = 2, *p* > .0001, question 1). The probability of growth was almost three times higher for contemporary nodules compared to historic nodules (contemporary probability = 0.968, historic probability = 0.359, χ^2^ = 82.82, df = 1, *p* > .0001, Figure 2, question 2). We also found that collection year (historic nodules only) and media type interacted to determine the probability of growth (χ^2^ = 21.17, df = 12, *p* = .04795, Figure 2d,g,h, question 3). Unfortunately, due to small samples for each media type, planned contrasts could not detect differences between media types, however it seems that isolates from 2004 did markedly worse on LB media, whereas those from 1950 had a slight preference for tryptone yeast.

### 3.2 Sanger sequencing

**FIGURE 3:**
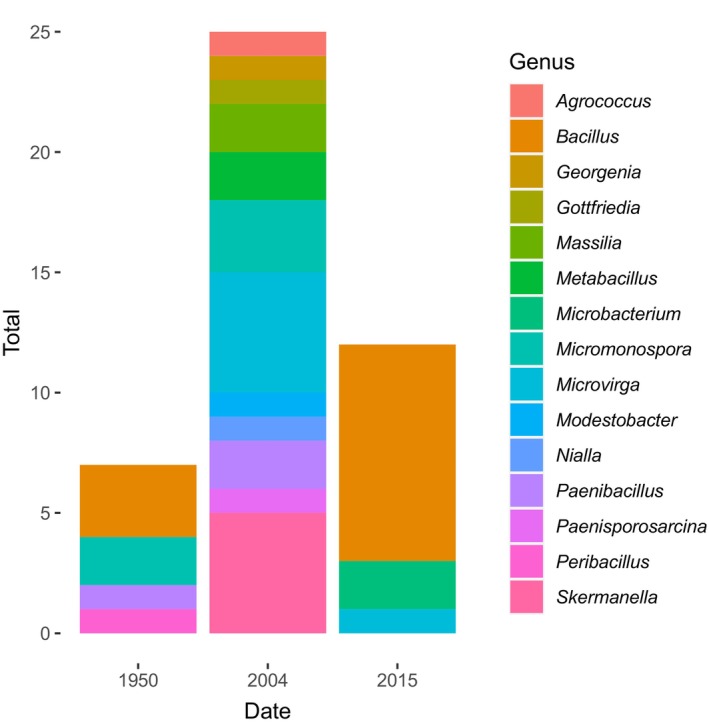
Sanger sequencing (done on 48% of our isolates) revealed isolates from four genera isolated from our 1950 sample. We identified isolates from 12 genera in our two nodules taken from the 2004 specimen. We found isolates from three genera from our nodule taken from a 2015 specimen.

In our sequenced samples, we identified 15 unique bacterial genera. Most isolates were in the genus *Bacillus* (~13% of our sequenced isolates) but a sizable amount belonged to other taxonomic groups including *Microvirga* (~6%), *Micromonospora* (~5%), *Skermanella* (~5%), and *Paenibacillus* (~3%). Isolates from four genera were isolated from our 1950 sample. We identified isolates from 12 genera in our two nodules taken from the 2004 specimen. We found isolates from three genera from our nodule taken from a 2015 specimen (Figure 3).

## 4 DISCUSSION

Here, we demonstrate that bacteria from historic herbarium specimens are culturable using microbiological media commonly used in microbiology research. Herbaria are potentially a powerful tool to study questions related to plant–microbe interactions, coevolution, and global change.

### 4.1 Potential nodule‐forming bacteria

Despite using media types (yeast mannitol) and conditions that are designed to culture rhizobia (Hungaria et al., 2016), we were not able to isolate *Ensifer* from any of our historic specimens, only from the contemporary *Medicago*. Although *Ensifer* are initially, highly numerically abundant the harsh selective conditions of the herbaria may decimate *Ensifer* populations. To prepare herbarium specimens for long‐term storage, plants are placed in a freezer to remove insect pests (−20°C to −60°C) and then moved to a drying cabinet (60°C) to reduce microbial activity (historically and occasionally still, depending on the herbarium, plants receive chemical treatments, e.g., mercuric chloride) (Woodland, 2009). The freezing alone is likely not problematic for *Ensifer*, but drying with force‐dry ovens can drastically reduce *Ensifer* viability (Vriezen et al., 2007), which allows for bacteria more robust to the unique conditions to persist. This is problematic when trying to isolate historic *Ensifer* but also creates an unusual opportunity to potentially detect nodule‐associated bacteria that would otherwise be hard to culture because of the dominance of *Ensifer*. Future work will include sequencing the rest of our culture collection and increasing our temporal resolution by sampling more specimens, from more dates, allowing us to definitively say how long *Ensifer* can survive in herbaria.

We were able to isolate potential nodule‐forming *Microvirga* from the nodules of specimens from 2004 and 2015. Nodule forming *Microvirga* form nodules in several legume species, including *Listia*, *Lupinus* (Ardley et al., 2012, 2013), and *Vigna* (Radl et al., 2014). *Microvirga* has not been described on *Medicago lupulina* but has been found in association with *Medicago sativa* nodules (Ilyas et al., 2022; Li et al., 2018) and the order Hyphomicrobiales is found in *Medicago polymorpha* nodules (Martínez‐Hidalgo et al., 2022). Additionally, 11 *Microvirga* OTUs were identified on *Medicago truncatula* nodules (Brown et al., 2020). In the future, we will investigate if historic *Microvirga* isolated from herbaria nodules can nodulate and benefit *Medicago lupulina*.

### 4.2 Non‐nodule forming bacteria

We were also able to recover a diversity of non‐nodule‐forming species of bacteria. The most abundant taxa in our collection are *Bacillus* and *Micromonospora*, two genera that are commonly found associated with nodules (De Meyer et al., 2015; Trujillo et al., 2010). *Bacillus* is potentially the most common nodule‐associated bacteria (Xu et al., 2014). Nodule‐associated *Bacillus* are important constituents of the nodule microbiome, solubilizing phosphate (Tapia‐García et al., 2020), promoting nodulation (Chinnaswamy et al., 2018), and enhancing plant size (Bai et al., 2002). Similarly, *Micromonospora* is commonly found in the nodule microbiome. In one study *Micromonospora* was found in 50% of *Trifolium* and *Medicago* nodules examined (Benito et al., 2017)*. Micromonospora* has been described as a rhizobia‐helper bacteria because of its ability to enhance nodulation and improve nitrogen use by plants (Martínez‐Hidalgo et al., 2014). In future work, would like to use assays to check for siderophore production (Ahmad et al., 2008), IAA production (Loper & Schroth, 1986), and phosphate solubilization (Ahmad et al., 2008). These functions are typically correlated with plant growth promotion. Finally, we will inoculate *Medicago lupulina* to check plant growth promotion with candidate strains. It is important to note that although we rinsed the outside of the nodule extensively (7 rinses) we did not bleach nodules. Some of the older nodules were slightly porous (confirmed under a dissecting scope), meaning that bleach could infiltrate the nodule. Because of this approach, it is possible that we cultured bacteria adhered tightly to the nodule exterior, rather than strict nodule endophytes. In addition, since we did not include root fragments we do not know if the bacteria we recovered are unique to nodules, they may be more generally associated with roots. However, since they were isolated from nodule tissue, we believe it is accurate to denote them as “nodule associated.” Future work should include culturing root fragments to differentiate nodule‐specific from general root‐associated bacteria.

### 4.3 The interesting case of the 1950 specimen

An outstanding question of this work is why we were able to culture from a 73‐year‐old specimen but not from specimens from intervening years (1974 and 1986). Because of our copious controls and rigorous experimental conditions, we believe that these isolates are not from environmental contamination. We suspect the reason we could culture from the 1950 specimen was one of two factors: (1) We had much higher sample sizes for the 1950 nodule. Initially, we thought the historic crushed nodule would provide enough material to streak 10 plates per media type, however it seemed there was not enough liquid so after streaking 10 plates of each media type for the 1950 nodule and its associated leaf control, we scaled the experiment back to three plates of each media type. This resulted in over three times as many plates for our 1950 specimen, this alone may have increased the probability that we could capture historic microbes. Additionally, (2) historic specimens are handled very differently depending on the collection conditions and/or the collector/curator at the herbarium (Forrest et al., 2019). Specimens are dried and frozen for variable amounts of time using different methods resulting in increased probability of microbial survival. We do not have information on how the 1950 specimen was handled but it is possible that it was handled in a way that allowed spore‐forming bacteria such as *Bacillus* (the dominant genus found in our collection) to persist. In our experiment collection year is confounded with specimen handling. To really understand how collection year influences culturability multiple specimens from the same year need to be used in a more rigorous design. In general, the collection we worked with was limited in both geographic and temporal scope. Our design could be improved upon by using larger collections with more specimens or using specimens from multiple herbaria.

### 4.4 Methodological/ethical considerations

We controlled for environmental contamination by rinsing nodules and including leaf controls, which presumably were exposed to the same sources of environmental contamination. However, post‐harvest plants have significantly different microbiomes than actively growing plants both in taxonomic diversity and function. This “post‐mortem” microbiome was found to be a significant portion of the microbes identified on herbarium specimens (*Alternaria alternata* comprised up to 7% of the total reads, Bieker et al., 2020). However, authors found that non‐bacterial microbes were the largest contributors of the leaf post‐mortem microbiome (Bieker et al., 2020). Potentially, some of the microbes that we cultured are constituents of *Medicago's* post‐mortem microbiome. We did isolate genera that are known constituents of the *Medicago* nodule microbiome including *Micromonospora* and *Paenibaccillus* (Hansen et al., 2020; Lai et al., 2015; Martínez‐Hidalgo et al., 2014). Further work should compare historic and contemporary nodule microbiomes of *Medicago lupulina* to determine the occurrence of taxa found in our collection in contemporary populations. Interestingly the number of genera we were able to recover is similar to the number of genera recovered in other nodule microbiome culturing work of both Medicago lupulina (14 genera, C. Chang personal communication, March 28, 2024) and other Medicago species (10 genera, Martínez‐Hidalgo et al., 2022).

General ethical issues that occur when using herbarium specimens are also relevant for this type of work. We took a highly conservative sampling approach because our approach requires destructively sampling specimens. We chose a common species that is abundant in herbaria across the country. It would be impractical and unethical to work with rare specimens that occur sporadically in herbaria. Fortunately, with the digitization of collections all over the country and world access to suitable specimens, this kind of work is increasingly possible. Finally, it is important to acknowledge that herbaria, like any biological collection, benefitted from the period of overt colonialism spanning the 15th to mid‐20th centuries. The majority of plant specimens are held in the global north, despite being collected globally, a pattern that has created an inverse relationship between global biodiversity and where collections are housed (Park et al., 2023). This trend in collections highlights not only a trend for the colonizing countries to exploit foreign resources but also wrests the control of knowledge and biological resources away from countries of origin, depriving resident explorers/scientists/researchers from local resources and discoveries.

## 5 FUTURE DIRECTIONS

A powerful approach will be to combine sequencing and culturing from herbarium specimens. These approaches together can provide a more holistic picture of the historical plant microbiome somewhat mitigating the limitations of each approach. Many microbes identified through sequencing are to date uncultured, and a major unknown in microbiology is how many will eventually be culturable (Steen et al., 2019). Limitations to culturing in general can present a biased/incomplete picture of the plant microbiome. Limitations to culturing herbarium microbes can further exacerbate this. Herbaria are a harsh selective environment where microbes experience selection due to specimen preparation, as well as extreme long‐term desiccation, which likely selects for microbes that create biofilms, produce trehalose, and form spores (Potfis, 1994).

Still, the culturable microbes open a range of possibilities for experimental work exploring microbial evolution, plant–microbe interactions, and coevolution. However, to get a more comprehensive understanding of the microbiome, this work could be done in conjunction with sequencing. Sequencing can provide an overview of the historical microbiome, however this approach is not without its limitations. The level of degradation of herbaria DNA classifies it as ancient DNA (Burbano & Gutaker, 2023), herbaria bacterial DNA experiences similar levels of degradation but is also prone to contamination issues because of the ubiquity of microbes on every surface and changes in the microbial community after mortality. Sequencing across herbaria may help deal with these issues by identifying and controlling for the post‐mortem microbiome found in herbaria (Bieker et al., 2020).

We believe this work has multiple applications, including bioprospecting, especially for stress‐tolerant microbes. Recent work found that bacteria exposed to dry conditions mitigated drought stress for plants, even if plants were not present during the stress event (Ricks & Yannarell, 2023). The intense selective conditions of the herbaria might be a novel source to bio‐prospect plant‐growth‐promoting bacteria, that can be used to mitigate drought stress for plants. Drought is currently one of the major impediments to agricultural productivity (Cotrina Cabello et al., 2023) and creative solutions are necessary in the face of climate change. This work also has interesting implications for conservation. It is widely acknowledged that animal and plant diversity is declining precipitously. However much less is known about microbial extinction (Thaler, 2021), and how this might be related to macro‐organism declines (Mueller et al., 2020). Sequencing from herbaria might open a window to understand past‐microbiome compositions and inoculating with microbes cultured from herbaria would allow researchers to directly test how extinct/rare microbes might facilitate plant restoration/conservation. Given the historically problematic nature of collections, using collections to restore biodiversity might be a particularly poetic next chapter in the use of herbaria, restoring important resources both to indigenous communities and to society at large.

## 6 CONCLUSIONS

Herbarium collections contain specimens that represent taxa from a wide range of locations collected over a timeframe that is well beyond that of modern research. Our work demonstrates that not only are herbarium specimens a source of genetic/genomic/metagenomic information about plant species and their associated microorganisms, but they are also the source of historic microbes. While microbial diversity has been examined extensively, microbial function is only now being explored. A source of data for understanding microbiome function is growing culture collections. Enhancing culture collections through herbarium collections will not only expand the source of culture collection material but might help identify novel functions that are relevant to pressing problems such as climate change (Rudgers et al., 2020). Additionally, herbarium microbial culture collections will allow us to test hypotheses about plant–microbe interactions over larger temporal and spatial scales.

## AUTHOR CONTRIBUTIONS

**Renee H. Petipas:** Conceptualization (equal); data curation (equal); formal analysis (equal); funding acquisition (equal); investigation (equal); methodology (equal); project administration (equal); validation (equal); visualization (equal); writing – original draft (equal); writing – review and editing (equal). **Amanda A. Antoch:** Conceptualization (equal); data curation (equal); investigation (equal); methodology (equal); validation (equal); writing – review and editing (equal). **Ashton A. Eaker:** Investigation (equal); methodology (equal); writing – review and editing (equal). **Hanna Kehlet‐Delgado:** Formal analysis (equal); methodology (equal); writing – review and editing (equal). **Maren L. Friesen:** Conceptualization (equal); funding acquisition (equal); resources (equal); supervision (equal); validation (equal); writing – review and editing (equal).

## CONFLICT OF INTEREST STATEMENT

The authors declare no conflict of interest.

## Supporting information


Appendix S1.

## Data Availability

Sequence data will be archived at GenBank® the National Institute of Health's genetic sequence database. Data files and R code can be found on the Open Science Framework (OSF) website: osfhttps://osf.io/qpykj/.
