# Supplementary material for: Back to the future: Using herbarium specimens to isolate nodule‐associated bacteria
Source: Ecol Evol. 2024 Jul 14;14(7):e11719. doi: 10.1002/ece3.11719 (PMC11246978; doi:10.1002/ece3.11719)
Supplement: Supplementary file 1 — Appendix S1. [file ECE3-14-e11719-s001.docx]

**Supplementary Information**

**Title:** Back to the future: Using herbarium specimens to isolate nodule associated bacteria

**Authors:**

Renee H. Petipas^1^

Amanda A. Antoch^1,2^

Ashton.N. Eaker^1^

Hanna Kehlet-Delgado^1^

Maren L. Friesen^1^

**Author Affiliations:**

^1^: Department of Plant Pathology, Washington State University, Pullman WA 99164

^2^:Department of Microbiology, University of Washington, Seattle WA

**Corresponding author:** Renee H. Petipas, renee.petipas@gmail.com

**Table S1: Recipes used to make the seven microbiological media types used in culturing historic and contemporary microbes from *Medicago lupulina* nodules. All amounts are in grams except for pyruvate (mM)**

| **Media component** | **Yeast mannitol** | **Tryptone yeast** | **Luria-Bertani** | **Dilute YM** | **YM+ Calcium** | **YM+ Salt** | **YM+ Pyruvate** |
| --- | --- | --- | --- | --- | --- | --- | --- |
|  |  |  |  |  |  |  |  |
| Yeast extract | 1 | 3 | 5 | 0.5 | 1 | 1 | 1 |
| Bactotryptone | 0 | 5 | 10 | 0 | 0 | 0 | 0 |
| Mannitol | 10 | 0 | 0 | 5 | 10 | 10 | 10 |
| Dipotassium phosphate | 0.5 | 0 | 0 | 0.25 | 0.5 | 0.5 | 0.5 |
| Magnesium sulfate | 0.2 | 0 | 0 | 0.1 | 0.2 | 0.2 | 0.2 |
| Sodium chloride | 0.1 | 0 | 10 | 0.05 | 0.1 | 1 | 0.1 |
| Calcium carbonate | 1 | 0 | 0 | 0.5 | 2 | 1 | 1 |
| Calcium chloride | 0 | 0.38 | 0 | 0 | 0 | 0 | 0 |
| Pyruvate | 0 | 0 | 0 | 0 | 0 | 0 | 2 |

| **Table S2: Table of isolate morphology, including the historical specimen bacteria were isolated from, a nodule ID (multiple isolates come from one nodule but are not confirmed as genetically unique). When we grew isolates up to confirm morphology and purity we were unable to get three isolates to grow and subsequently they have no description included here.** | | | | | | | | | | | | |
| --- | --- | --- | --- | --- | --- | --- | --- | --- | --- | --- | --- | --- |
| **Specimen** | **Nodule** | **Year** | [**Plate ID**](http://plate.id/) | [**Isolate**](http://strain.id/) | **Species ID** | **Plate type** | **Pigmentation** | **Opacity** | **Form** | **Margin** | **Elevation** | **Texture** |
| 390849 | CF7225 | 2004 | CF7225--164 | CF3001 | NA | TY | white pink | clear | irregular | entire | pulvinate | mucoid |
| 390849 | CF7225 | 2004 | CF7225--167 | CF3002 | NA | TY | white pink | clear | irregular | filiform | flat | dry matte |
| 390849 | CF7225 | 2004 | CF7225--513 | CF3003 | Microvirga | YM+NaCl | pink | translucent | irregular | entire | pulvinate | mucoid |
| 390849 | CF7225 | 2004 | CF7225--743 | CF3004 | NA | YM+Pyr | white | clear | rhizoid | filiform | flat | dry matte |
| 390614 | CF7226 | 2015 | CF7226--063 | CF3005 | Bacillus | YM | beige | opaque | irregular | erose | flat | moist matte |
| 390614 | CF7226 | 2015 | CF7226--064 | CF3006 | NA | YM | beige | opaque | irregular | erose | flat | moist matte |
| 390614 | CF7226 | 2015 | CF7226--065 | CF3007 | Poor quality | YM | beige | opaque | irregular | erose/curled | flat | moist matte |
| 390614 | CF7226 | 2015 | CF7226--175 | CF3008 | NA | TY | beige | opaque | irregular | erose/curled | flat | moist matte |
| 390614 | CF7226 | 2015 | CF7226--176 | CF3009 | Bacillus | TY | beige | opaque | irregular | erose/curled | flat | moist matte |
| 390614 | CF7226 | 2015 | CF7226--287 | CF3010 | Microbacterium | LB | neon yellow | opaque | circular | undulate | convex | moist |
| 390614 | CF7226 | 2015 | CF7226--291 | CF3011 | Bacillus | LB | beige | opaque | irregular | undulate | flat | dry bumpy |
| 390614 | CF7226 | 2015 | CF7226--292 | CF3012 | NA | LB | beige | opaque | irregular | erose | flat | moist matte |
| 390614 | CF7226 | 2015 | CF7226--408 | CF3013 | NA | DiluteYM | beige | opaque | irregular | erose/curled | flat | dry matte |
| 390614 | CF7226 | 2015 | CF7226--409 | CF3014 | Bacillus | DiluteYM | beige | opaque | irregular | curled | flat | moist matte |
| 390614 | CF7226 | 2015 | CF7226--523 | CF3015 | NA | YM+NaCl | beige | opaque | irregular | erose/curled | flat | dry bumpy matte |
| 390614 | CF7226 | 2015 | CF7226--525 | CF3016 | Microvirga | YM+NaCl | white pink | clear | circular | entire | raised | mucoid |
| 390614 | CF7226 | 2015 | CF7226--636 | CF3017 | NA | YM+Ca | beige | opaque | irregular | erose/curled | flat | moist bumpy matte |
| 390614 | CF7226 | 2015 | CF7226--637 | CF3018 | NA | YM+Ca | beige | opaque | irregular | erose/curled | flat | moist bumpy matte |
| 390614 | CF7226 | 2015 | CF7226--638 | CF3019 | NA | YM+Ca | beige | opaque | irregular | erose/curled | flat | dry matte |
| 390614 | CF7226 | 2015 | CF7226--753 | CF3020 | Bacillus | YM+Pyr | beige | opaque | irregular | erose/curled | flat | moist |
| 390614 | CF7226 | 2015 | CF7226--754 | CF3021 | NA | YM+Pyr | beige | opaque | irregular | erose/curled | flat | moist bumpy wrinkly |
| 390614 | CF7226 | 2015 | CF7226--755 | CF3022 | NA | YM+Pyr | beige | opaque | irregular | erose/curled | flat | dry |
| 390849 | CF7227 | 2004 | CF7227--070 | CF3023 | NA | YM | white pink | clear | circular | entire | pulvinate | mucoid |
| 390849 | CF7227 | 2004 | CF7227--184 | CF3024 | NA | TY | beige yellow tint | opaque | irregular | erose/curled | flat | moist bumpy |
| 390849 | CF7227 | 2004 | CF7227--185 | CF3025 | NA | TY | grey | translucent | irregular | erose | flat | moist |
| 390849 | CF7227 | 2004 | CF7227--305 | CF3026 | NA | LB | beige | opaque | circular | erose | raised | dry bumpy matte |
| 390849 | CF7227 | 2004 | CF7227--418 | CF3027 | NA | DiluteYM |  |  |  |  |  |  |
| 390849 | CF7227 | 2004 | CF7227--535 | CF3028 | NA | YM+NaCl | yellow | clear | irregular | lobate | raised | moist bumpy |
| 390849 | CF7227 | 2004 | CF7227--644 | CF3029 | NA | YM+Ca | pink | clear | circular | entire | raised | mucoid |
| 390849 | CF7227 | 2004 | CF7227--764 | CF3030 | NA | YM+Pyr | white yellow green | clear | circular | entire | raised edge | moist |
| 291521 | CF7229 | 1950 | CF7229--141 | CF3031 | NA | TY | beige | translucent | circular | lobate | umbonate | moist |
| 291521 | CF7229 | 1950 | CF7229--142 | CF3032 | Bacillus | TY | beige | translucent | irregular | erose | pulvinate | mucoid matte |
| 291521 | CF7229 | 1950 | CF7229--143 | CF3033 | Bacillus | TY | beige | opaque | irregular | entire | flat | dry bumpy matte |
| 291521 | CF7229 | 1950 | CF7229--144 | CF3034 | NA | TY | brown | opaque | irregular | curled | raised | moist bumpy |
| 291521 | CF7229 | 1950 | CF7229--265 | CF3035 | Peribacillus | LB | brown | opaque | irregular | curled | raised | moist |
| NA | CF7208 | 2018 | CF7208--016 | CF3036 | Bacillus | YM | white | clear | irregular | entire | pulvinate | mucoid |
| NA | CF7208 | 2018 | CF7208--592 | CF3040 | Sinorhizobium | YM+Ca | white green tint | clear | irregular | entire | pulvinate | mucoid |
| NA | CF7230 | 2019 | CF7230--253 | CF3044 | Bacillus | LB | beige | opaque | irregular | undulate | flat | dry matte |
| NA | CF7230 | 2019 | CF7230--368 | CF3045 | Poor quality | DiluteYM | white yellow green tint | clear | punctiform | entire | raiseed | moist |
| NA | CF7230 | 2019 | CF230--711 | CF3048 | NA | YM+Pyr | beige green tint | opaque | irregular | erose | flat | dry matte |
| NA | NA | Lab | WSM419--703 | CF3055 | Sinorhizobium | YM+Pyr | white | clear | irregular | entire | pulvinate | mucoid |
| 390614 | CF7226 | 2015 | CF7226--408 | CF3056 | Bacillus | DiluteYM | beige | opaque | irregular | curled | flat | dry matte |
| 390614 | CF7226 | 2015 | CF7226--409 | CF3057 | NA | DiluteYM | beige | opaque | irregular | erose/curled | flat | moist bumpy matte |
| 390614 | CF7226 | 2015 | CF7226--065 | CF3058 | NA | YM | beige | opaque | irregular | erose/curled | flat | dry bumpy matte |
| 390614 | CF7226 | 2015 | CF7226--063 | CF3059 | NA | YM | beige | opaque | circular | erose/undulate | raised | moist bumpy matte |
| 390614 | CF7226 | 2015 | CF7226--636 | CF3061 | Bacillus | YM+Ca | beige | opaque | irregular | erose/undulate | flat | matte |
| 390849 | CF7227 | 2004 | CF7227--644 | CF3062 | Microvirga | YM+Ca | hot pink | opaque | punctiform | entire | raised | moist |
| 390849 | CF7225 | 2004 | CF7225--513 | CF3064 | Nialla | YM+NaCl | white | translucent | filamentous | lobate | flat | dry matte |
| 390849 | CF7227 | 2004 | CF7227--535 | CF3065 | NA | YM+NaCl | white green tint | translucent | irregular | lobate | flat | matte |
| 390849 | CF7225 | 2004 | CF7225--743 | CF3066 | NA | YM+Pyr | grey | transparent | irregular | lobate | flat | moist bumpy matte |
| 390614 | CF7226 | 2015 | CF7226--753 | CF3067 | Microbacterium | YM+Pyr | neon yellow | opaque | punctiform | entire | raised | dry matte |
| 291521 | CF7229 | 1950 | CF7229--143 | CF3068 | NA | TY | brown | opaque | irregular | entire | concave | moist bumpy |
| 291521 | CF7229 | 1950 | CF7229--143 | CF3069 | Paenibacillus | TY | white | translucent | irregular | entire | raised | mucoid |
| 291521 | CF7229 | 1950 | CF7229--144 | CF3070 | NA | TY | white | translucent | irregular | entire | raised | mucoid |
| 390849 | CF7227 | 2004 | CF7227--756 | CF3085 | NA | YM+Pyr | white green tint | clear | punctiform | entire | flat | moist |
| 390849 | CF7227 | 2004 | CF7227--756 | CF3087 | Massilia | YM+Pyr | yellow beige | opaque | circular | entire | umbonate | moist |
| 390849 | CF7225 | 2004 | CF7225--744 | CF3088 | Metabacillus | YM+Pyr | beige pink | opaque | circular | curled | convex | moist bumpy matte |
| 390849 | CF7225 | 2004 | CF7225--744 | CF3089 | Metabacillus | YM+Pyr | beige | opaque | circular | curled | convex | moist bumpy matte |
| 390849 | CF7225 | 2004 | CF7225--745 | CF3091 | NA | YM+Pyr | yellow | translucent | circular | entire | convex | moist |
| 390849 | CF7225 | 2004 | CF7225--745 | CF3092 | NA | YM+Pyr | orange | opaque | punctiform | entrie | convex/under | dry |
| 291521 | CF7229 | 1950 | CF7229--720 | CF3093 | Micromonospora | YM+Pyr | orange | opaque | punctiform | entire | convex/under | dry |
| 291521 | CF7229 | 1950 | CF7229--722 | CF3094 | NA | YM+Pyr | orange | opaque | punctiform | entire | convex/under | dry |
| 390849 | CF7225 | 2004 | CF7225--399 | CF3098 | NA | DiluteYM | white pink | clear | circular | entire | raised | mucoid |
| 390849 | CF7225 | 2004 | CF7225--399 | CF3100 | NA | DiluteYM | white pink | clear | punctiform | entire | raised | mucoid |
| 390849 | CF7227 | 2004 | CF7227--419 | CF3101 | NA | DiluteYM | white pink | clear | punctiform | entire | pulvinate | mucoid |
| 390849 | CF7227 | 2004 | CF7227--419 | CF3102 | Paenisporosarcina | DiluteYM | white beige green tint | opaque | circular | erose/curled | convex | moist bumpy matte |
| 390849 | CF7227 | 2004 | CF7227--419 | CF3104 | NA | DiluteYM | white beige green tint | opaque | circular | entire | raised | moist mucoid |
| 390849 | CF7227 | 2004 | CF7227--419 | CF3105 | NA | DiluteYM | pink | clear | circular | entire | pulvinate | mucoid |
| 291521 | CF7229 | 1950 | CF7229--378 | CF3106 | Micromonospora | DiluteYM | orange | opaque | circular | entire | convex/under | dry matte |
| 390849 | CF7225 | 2004 | CF7225--284 | CF3108 | Georgenia | LB | colorless | clear | irregular | entire | raised | mucoid |
| 291521 | CF7229 | 1950 | CF7229--258 | CF3109 | Bacillus | LB | beige | opaque | irregular | erose/undulate | raised | moist bumpy |
| 390849 | CF7227 | 2004 | CF7227--532 | CF3110 | Gottfriedia | YM+NaCl | colorless | clear | circular | lobate | raised | moist |
| 390849 | CF7227 | 2004 | CF7227--532 | CF3111 | NA | YM+NaCl | hot pink | opaque | punctiform | entire | flat | dry |
| 390849 | CF7227 | 2004 | CF7227--533 | CF3113 | NA | YM+NaCl | white | clear | irregular | entire | pulvinate | mucoid |
| 390849 | CF7227 | 2004 | CF7227--533 | CF3114 | Skermanella | YM+NaCl | white pink | clear | irregular | entire | pulvinate | mucoid |
| 390849 | CF7225 | 2004 | CF7225--512 | CF3115 | NA | YM+NaCl | orange | opaque | punctiform | entire | convex/under | dry |
| 390849 | CF7225 | 2004 | CF7225--512 | CF3116 | Agrococcus | YM+NaCl | yellow | translucent | irregular | entire | raised | moist |
| 390849 | CF7225 | 2004 | CF7225--512 | CF3117 | NA | YM+NaCl | white green tint | clear | irregular | entire | raised | mucoid |
| 390849 | CF7229 | 1950 | CF7229--487 | CF3119 | NA | YM+NaCl | beige | clear | circular | erose/curled | flat | moist |
| 390849 | CF7225 | 2004 | CF7225--627 | CF3120 | Micromonospora | YM+Ca | orange | opaque | punctiform | entire |  |  |
| 390849 | CF7225 | 2004 | CF7225--628 | CF3121 | NA | YM+Ca | white | opaque | circular | entire | umbonate | moist |
| 390849 | CF7225 | 2004 | CF7225--628 | CF3122 | NA | YM+Ca | beige | opaque | circular | erose/curled | raised | moist matte |
| 390849 | CF7225 | 2004 | CF7225--164 | CF3123 | Skermanella | TY | white | clear | irregular | entire | pulvinate | mucoid |
| 390849 | CF7225 | 2004 | CF7225--164 | CF3124 | NA | TY | white pink | clear | irregular | entire | pulvinate | mucoid |
| 390849 | CF7225 | 2004 | CF7225--626 | CF3125 | NA | YM+Ca | orange | opaque | punctiform | entire | convex/under | dry |
| 390849 | CF7225 | 2004 | CF7225--626 | CF3126 | Skermanella | YM+Ca | beige pink | clear | irregular | entire | pulvinate | mucoid |
| 390849 | CF7225 | 2004 | CF7225--626 | CF3127 | Modestobacter | YM+Ca | black green | punctiform | punctiform | undulate | clumpy | dry powdery |
| 390849 | CF7225 | 2004 | CF7225--626 | CF3129 | NA | YM+Ca | beige pink | clear | irregular | entire | pulvinate | mucoid |
| 390849 | CF7227 | 2004 | CF7227--646 | CF3130 | Microvirga | YM+Ca | neon yellow | opaque | circular | entire | raised | moist |
| 390849 | CF7227 | 2004 | CF7227--646 | CF3131 | Massilia | YM+Ca |  |  |  |  |  |  |
| 390849 | CF7227 | 2004 | CF7227--646 | CF3132 | Poor quality | YM+Ca | white | opaque | circular | entire | pulvinate | mucoid |
| 390849 | CF7227 | 2004 | CF7227--646 | CF3133 | NA | YM+Ca |  |  |  |  |  |  |
| 390849 | CF7227 | 2004 | CF7227--075 | CF3134 | Poor quality | YM | white pink | translucent | irregular | entire | raised | moist |
| 390849 | CF7227 | 2004 | CF7227--075 | CF3135 | NA | YM | white pink | translucent | irregular | entire | pulvinate | mucoid |
| 390849 | CF7227 | 2004 | CF7227--075 | CF3136 | Skermanella | YM | beige pink | clear | irregular | entire | pulvinate | mucoid |
| 390849 | CF7225 | 2004 | CF7225--054 | CF3137 | Microvirga | YM | white beige | clear | irregular | entire/undulate | raised | mucoid |
| 390849 | CF7225 | 2004 | CF7225--055 | CF3138 | NA | YM | white pink tint | opaque | irregular | entire/undulate | raised | mucoid |
| 390849 | CF7225 | 2004 | CF7225--053 | CF3140 | Skermanella | YM | beige pink | clear | irregular | entire | pulvinate | mucoid |
| 390849 | CF7225 | 2004 | CF7225--054 | CF3141 | Micromonospora | YM | orange | opaque | punctiform | entire | convex/under | dry |
| 390849 | CF7225 | 2004 | CF7225--165 | CF3142 | Micromonospora | TY | orange | opaque | punctiform | entire | convex/under | dry |
| 390614 | CF7226 | 2015 | CF7226--525 | CF3144 | Bacillus | YM+NaCl | beige | opaque | irregular | erose/curled | raised | moist wrinkly matte |
| 390849 | CF7225 | 2004 | CF7225--055 | CF3146 | NA | YM | orange | opaque | punctiform | entire | convex/under | dry |
| 390849 | CF7225 | 2004 | CF7225--628 | CF3147 | Paenibacillus | YM+Ca | white | opaque | circular | curled | umbonate | dry |
| 390849 | CF7227 | 2004 | CF7227--644 | CF3149 | NA | YM+Ca | white | clear | punctiform | entire | flat | moist |
| 390614 | CF7226 | 2015 | CF7226--753 | CF3150 | Bacillus | YM+Pyr | beige | opaque | irregular | erose/curled | raised | moist wrinkly matte |
| 390849 | CF7225 | 2004 | CF7225--399 | CF3153 | Microvirga | DiluteYM | beige pink | clear | irregular | entire | pulvinate | mucoid |
| 390849 | CF7227 | 2004 | CF7227--070 | CF3155 | Paenibacillus | YM | beige | clear | irregular | lobate/undulate | raised | moist |

**Table S3: Blast search results for Sanger sequenced isolates collected from contemporary (2018, 2019, Lab) and historic (1950, 2004, 2015) *Medicago lupulina.* Query seq type refers to if the entire assembled sequence was blasted (27f/1492r ), one, or both primers (indicated by a bolded box). Hit accession is the National Center for Biotechnology Information accession number. Percent pairwise identity is listed in the %PI column. The bit-score describes the quality of the alignment while the E-value describes the number of hits that could be found by chance alone. High bit scores (>50) and low E-values indicate high quality matches.**

| Isolate ID | Year | Query seq type | Top hit ID Description | Hit Accession | % PI | Bit-Score | E Value |
| --- | --- | --- | --- | --- | --- | --- | --- |
|  |  |  |  |  |  |  |  |
| CF3003 | 2004 | 27f/1492r | Microvirga makkahensis strain SV1470 | NR_149218 | 98.00% | 2335.29 | 0 |
| CF3005 | 2015 | 27f/1492r | Bacillus wiedmannii strain FSL W8-0169 | NR_152692 | 99.50% | 2567.96 | 0 |
| CF3009 | 2015 | 27f/1492r | Bacillus wiedmannii strain FSL W8-0169 | NR_152692 | 99.20% | 2538.42 | 0 |
| CF3010 | 2015 | 27f/1492r | Microbacterium yannicii strain G72 | NR_117001 | 98.10% | 2447.93 | 0 |
| CF3011 | 2015 | 27f/1492r | Bacillus wiedmannii strain FSL W8-0169 | NR_152692 | 99.20% | 2551.34 | 0 |
| CF3014 | 2015 | 27f/1492r | Bacillus cereus ATCC 14579 | NR_074540 | 99.50% | 2577.2 | 0 |
| CF3016 | 2015 | 27f/1492r | Microvirga lupini strain Lut6 | NR_115984 | 97.90% | 2381.45 | 0 |
| CF3020 | 2015 | 1492r | Bacillus wiedmannii strain FSL W8-0169 | NR_152692 | 98.80% | 1559.69 | 0 |
| CF3032 | 1950 | 1492r | Bacillus atrophaeus strain NBRC 15539 | NR_112723 | 99.60% | 1482.13 | 0 |
| CF3033 | 1950 | 27f/1492r | Bacillus mojavensis strain NBRC 15718 | NR_112725 | 99.80% | 2590.12 | 0 |
| CF3035 | 1950 | 27f/1492r | Peribacillus frigoritolerans strain DSM 8801 | NR_117474 | 99.60% | 2606.74 | 0 |
| CF3036 | 2018 | 27f/1492r | Bacillus wiedmannii strain FSL W8-0169 | NR_152692 | 99.40% | 2584.58 | 0 |
| CF3040 | 2018 | 27f/1492r | Sinorhizobium medicae strain 11-3 21a | NR_104719 | 99.70% | 2464.55 | 0 |
| CF3044 | 2019 | 1492r | Bacillus wiedmannii strain FSL W8-0169 | NR_152692 | 99.60% | 1423.04 | 0 |
| CF3055 | Lab | 27f/1492r | Sinorhizobium medicae strain 11-3 21a | NR_104719 | 99.40% | 2486.71 | 0 |
| CF3056 | 2015 | 27f | Bacillus paramycoides strain MCCC 1A04098 | NR_157734 | 99.10% | 893.052 | 0 |
| CF3056 | 2015 | 1492r | Bacillus wiedmannii strain FSL W8-0169 | NR_152692 | 99.30% | 1098.03 | 0 |
| CF3061 | 2015 | 27f/1492r | Bacillus wiedmannii strain FSL W8-0169 | NR_152692 | 99.20% | 2571.66 | 0 |
| CF3062 | 2004 | 27f | Microvirga soli strain R491 | NR_156051 | 99.00% | 776.713 | 0 |
| CF3062 | 2004 | 1492r | Microvirga soli strain R491 | NR_156051 | 98.70% | 1208.83 | 0 |
| CF3064 | 2004 | 1492r | Niallia nealsonii strain DSM 15077 | NR_044546 | 98.00% | 1500.6 | 0 |
| CF3067 | 2015 | 27f/1492r | Microbacterium yannicii strain G72 | NR_117001 | 96.40% | 2326.05 | 0 |
| CF3069 | 1950 | 27f/1492r | Paenibacillus glycanilyticus strain DS-1 | NR_024759 | 99.00% | 2518.1 | 0 |
| CF3087 | 2004 | 27f/1492r | Massilia agri strain K-3-1 | NR_157781 | 98.40% | 1727.74 | 0 |
| CF3088 | 2004 | 27f | Metabacillus idriensis strain SMC 4352-2 | NR_043268 | 99.40% | 1044.48 | 0 |
| CF3088 | 2004 | 1492r | Metabacillus idriensis strain SMC 4352-2 | NR_043268 | 99.30% | 1410.11 | 0 |
| CF3089 | 2004 | 27f/1492r | Metabacillus idriensis strain SMC 4352-2 | NR_043268 | 99.10% | 2466.4 | 0 |
| CF3093 | 1950 | 27f | Micromonospora zamorensis strain CR38 | NR_108479 | 98.00% | 1086.95 | 0 |
| CF3102 | 2004 | 27f | Paenisporosarcina macmurdoensis strain CMS 21w | NR_025573 | 99.00% | 1260.54 | 0 |
| CF3102 | 2004 | 1492r | Paenisporosarcina macmurdoensis strain CMS 21w | NR_025573 | 99.20% | 1199.6 | 0 |
| CF3106 | 1950 | 27f/1492r | Micromonospora phytophila strain SG15 | NR_159268 | 98.50% | 2386.99 | 0 |
| CF3108 | 2004 | 27f | Georgenia soli strain CC-NMPT-T3 | NR_116959 | 98.80% | 813.646 | 0 |
| CF3109 | 1950 | 1492r | Bacillus chungangensis strain CAU 348 | NR_116709 | 98.70% | 1400.88 | 0 |
| CF3110 | 2004 | 27f/1492r | Gottfriedia solisilvae strain NEAU-cbsb5 | NR_159143 | 99.00% | 2531.03 | 0 |
| CF3114 | 2004 | 1492r | Skermanella aerolata strain 5416T-32 | NR_043929 | 98.70% | 789.64 | 0 |
| CF3116 | 2004 | 27f/1492r | Agrococcus jenensis strain DSM 9580 | NR_026275 | 97.90% | 2411 | 0 |
| CF3120 | 2004 | 1492r | Micromonospora zamorensis strain CR38 | NR_108479 | 99.70% | 1007.54 | 0 |
| CF3123 | 2004 | 27f | Skermanella aerolata strain 5416T-32 | NR_043929 | 99.00% | 1127.58 | 0 |
| CF3126 | 2004 | 27f | Skermanella aerolata strain 5416T-32 | NR_043929 | 99.20% | 828.419 | 0 |
| CF3127 | 2004 | 27f/1492r | Modestobacter caceresii strain KNN 45-2b | NR_137398 | 98.70% | 2409.15 | 0 |
| CF3130 | 2004 | 27f | Microvirga ossetica strain V5/3M | NR_156049 | 97.20% | 843.193 | 0 |
| CF3130 | 2004 | 1492r | Microvirga pakistanensis strain NCCP-1258 | NR_152637 | 98.30% | 990.925 | 0 |
| CF3131 | 2004 | 27f/1492r | Massilia agri strain K-3-1 | NR_157781 | 98.30% | 2444.24 | 0 |
| CF3136 | 2004 | 27f/1492r | Skermanella aerolata strain 5416T-32 | NR_043929 | 99.30% | 2436.85 | 0 |
| CF3137 | 2004 | 27f/1492r | Microvirga makkahensis strain SV1470 | NR_149218 | 98.30% | 2368.53 | 0 |
| CF3140 | 2004 | 1492r | Skermanella aerolata strain 5416T-32 | NR_043929 | 99.30% | 1325.17 | 0 |
| CF3141 | 2004 | 27f/1492r | Micromonospora soli strain SL3-70 | NR_146360 | 98.20% | 2351.91 | 0 |
| CF3142 | 2004 | 27f/1492r | Micromonospora luteifusca strain GUI2 | NR_137397 | 98.40% | 2375.91 | 0 |
| CF3144 | 2015 | 27f/1492r | Bacillus wiedmannii strain FSL W8-0169 | NR_152692 | 99.40% | 2564.27 | 0 |
| CF3147 | 2004 | 1492r | Paenibacillus pocheonensis strain Gsoil 1138 | NR_112565 | 98.00% | 1040.78 | 0 |
| CF3150 | 2015 | 27f/1492r | Bacillus cereus ATCC 14579 | NR_074540 | 99.50% | 2595.66 | 0 |
| CF3153 | 2004 | 27f/1492r | Microvirga makkahensis strain SV1470 | NR_149218 | 98.50% | 2287.27 | 0 |
| CF3155 | 2004 | 27f/1492r | Paenibacillus humicus strain PC-147 | NR_042577 | 98.50% | 2477.48 | 0 |
